# Working hours and health – key research topics in the past and future

**DOI:** 10.5271/sjweh.4157

**Published:** 2024-05-01

**Authors:** Mikko Härmä, Göran Kecklund, Philip Tucker

**Affiliations:** 1 Finnish Institute of Occupational Health (FIOH), Work Ability and Work Careers, Helsinki, Finland.; 2 Department of Psychology, Division of Psychobiology and Epidemiology, Stockholm University, Sweden.; 3 School of Psychology, Swansea University, Swansea, Wales, United Kingdom.; 4 Department of Psychology, Division of Psychobiology and Epidemiology, Stockholm University, Sweden.

**Keywords:** long working hour, mechanism, research agenda, safety, shift work, study design

## Abstract

**Objective:**

This paper discusses the past and present highlights of working hours and health research and identifies key research needs for the future.

**Method:**

We analyzed over 220 original articles and reviews on working hours and health in the *Scandinavian Journal of Work, Environment & Health* published during the last 50 years. Key publications from other journals were also included.

**Results:**

The majority of identified articles focussed on the effects of shift and night work, with fewer studying long and reduced working hours and work time control. We observed a transition from small-scale experimental and intensive field studies to large-scale epidemiological studies utilizing precise exposure assessment, reflecting the recent emergence of register-based datasets and the development of analytic methods and alternative study designs for randomized controlled designs. The cumulative findings provide convincing evidence that shift work and long working hours, which are often associated with night work and insufficient recovery, increase the risk of poor sleep and fatigue, sickness absence, occupational injuries, and several chronic health conditions such as cardiovascular diseases and cancer. The observed risks are strongly modified by individual and work-related factors.

**Conclusions:**

Although the observed health risks of shift work and long working hours are mostly low or moderate, the widespread prevalence of exposure and the hazardousness of the many associated potential outcomes makes such working time arrangements major occupational health risks. Further research is needed to identify exposure–response associations, especially in relation to the chronic health effects, and to elucidate underlying pathways and effective personalized intervention strategies.

The *Scandinavian Journal of Work, Environment & Health* has published over 220 scientific articles on working time and health over the 50 years of its existence. These include original research but also around 30 reviews. Altogether, shift work and working hours were the main reported exposures in 11% of the papers published between 1975 and 2023 (1). From 1975 to 1984, shift work and working hours was the eighth most common topic in the Journal, whereas from 2015 to 2023, it has become the second most popular topic after psychosocial work environment. Working hours and health scores high in the Journal's citation rankings. For example, a review on shift work and cardiovascular diseases (2) and two reviews on long working hours and health (3, 4) are among the ten most cited reviews of the Journal’s 50 years (1).

In this paper, we discuss the past and present highlights of working hours and health research and identify some key research needs for the future. We analyzed 220 scientific articles on working hours and health published in the *Scandinavian Journal of Work, Environment & Health*. Key publications on working hours and health from other journals were also considered.

## Circadian rhythms in shift work

Based on animal and human studies in time-isolated “bunkers”, Franz Halberg, Jürgen Aschoff and Rütger Wever built the theoretical framework and basis of “circadian physiology” in the 1950s and 1960s. While epidemiological research on shift work and health had appeared sporadically before this, this basic research on chronobiology gave birth to the study of night and shift work as we know it today.

The first chronobiological studies among shift workers focused on the circadian variation of physiological and cognitive functions during nights shifts. Fifty years ago, a field study by Kolmodin-Hedman on 132 Swedish railway workers (5) reported that the amount of sleep during night work was significantly less than it was for day work or on days off. Body temperature and other circadian rhythms followed a day-oriented pattern indicating non-adaption to the inverse sleep-wake rhythm of night work. In another study, the circadian rhythms of male typesetters also showed only partial adaption to shift work, even after a 3-year follow-up (6). Later studies showed that even permanent night work is unlikely to result in a degree of circadian adjustment that could be regarded as sufficient adaption to night work (7).

## Laboratory and field studies

Studies of circadian rhythms in shift work in naturalistic studies could not answer the practical question of how to optimize the organization of night and shift work. Based on a symposium in Dortmund, Germany, the two pioneers of shift work research, Joseph Rutenfranz and Peter Colquhoun announced: “We need much more information based on experimental studies of shift work before we can feel fully confident in advising industrial and other organizations about their own particular problems in the area.” (8) The new research questions focused on adaption to night and shift work in relation to the characteristics of the rotation systems (8, 9). The results suggested that rapidly rotating (2–3 consecutive night shifts) and clockwise-rotating shift systems (when changes from one type of shift to the next involve delaying the timing of sleep – eg, morning shifts – followed by evening shifts and then night shifts) were preferable compared to more slowly and counter-clockwise-rotating shift systems (10–12).

Many studies in the 1990s focused on the effects of long work shifts. For example, an intervention study conducted over ten months showed that 12-hour shifts were linked to larger decrements in sleep length, alertness and performance, compared to 8-hour shifts (13). Later, in a rare randomized controlled trial, police officers were allocated to schedules comprising either 8-, 10- or 12-hour shifts (14). The results favored the use of 10-hour shifts over the traditional 8-hour shifts but did not favor the longer 12-hour shifts. Unlike this study, most early intervention studies on shift work and long work shifts were non-randomized before–after studies, involving the simultaneous change of two or more shift characteristics (eg, shift length and the direction of shift rotation), making it difficult to conclude on the benefits of any individual characteristic (12).

More recently, a considerable amount of shift work research has focused on recovery time between shifts, with the inter-shift interval being closely related to the direction of rotation. Forward-rotating schedules (including 12-hour shift systems) normally have ≥24 hours between successive shifts, while backward-rotating systems have more “quick returns”, ie, ≤11 hours between the shifts. In field studies, quick returns are associated with shorter sleep and higher sleepiness (15), consistent with the observed benefits of forward (clockwise) rotating shifts.

## Sleep, fatigue and injuries

One of the most researched topics within the field of working time research is the role that circadian rhythms play in the regulation of sleep and fatigue in shift work. Sleep duration shows a pronounced time-of-day pattern with the shortest sleeps being those initiated during daytime. Sleep-related fatigue (which is often labelled sleepiness) also shows a circadian pattern, with elevated levels during night time and a peak in the early morning hours (16). Furthermore, fatigue increases during the acute and repeated sleep deprivation that is associated with, for example, morning and night work. [Night shifts are normally defined as shifts lasting ≥3 hours between 23:00 and 06:00 hours (17, 18), while morning, day and evening shifts are defined according to their starting times. Morning shift start typically after 06:00 but those starting 03:00–06:00 are called “early morning shifts” (19)].

In the 1980s, studies corroborated earlier findings that both night and morning work are associated with short-sleep durations, using objective sleep recording carried out in the homes of the participants. In a field study by Torsvall and coworkers (20), the shortest sleep durations (approximately 5.5 hours) were observed in connection with early morning and night work, whereas sleep duration was >2 hours longer for afternoon shifts and day work (ie, conventional “9 to 5” working days). Another such field study observed that the shortening of sleep duration during morning and night work resulted in less REM, stage 2, and somewhat less deep sleep (21). With regards to subjective sleep quality, the study by Åkerstedt et al (21) showed that in 3-shift work, the morning shift in particular was associated with disturbed sleep, as indicated by non-refreshing sleep and difficulties waking up.

One of the consequences of the sleep restriction, fatigue and sleepiness that are associated with night and shift work is the increased risk of occupational injuries. Several studies published in this Journal have observed increased risk of injuries during night work (22–26) and on work days following night shifts (27). Similarly, several studies have found elevated risk of occupational injuries on evening shifts (22, 25, 27), suggesting that other work shift-related differences, like higher individual work load in addition to sleep restriction, may contribute to the increased injury risk.

The negative consequences of fatigue due to quick returns have been highlighted in several recent studies showing that injury risk increased following these short inter-shift intervals (27–29). Further support for the link between shift schedule design and injuries was found in an intervention study that compared users and non-users of a shift scheduling tool that provided scheduling recommendations (26). The group that used the tool obtained longer recovery periods (eg, less quick returns, more single days off) and there was a reduction in the incidence of occupational injuries.

## Individual differences in shift work tolerance

The previously mentioned study by Torsvall and colleagues (20) provided an early demonstration that night work may become more problematic with increasing age. The findings revealed that night work had a more detrimental effect on the sleep of the older workers, compared to their younger counterparts. Further evidence of age-related differences in shiftwork tolerance was provided by a simulated shift work study of postal workers sorting letters that featured physiological and behavioural measures of circadian timing and sleep (30). It showed that older age was associated with less circadian adjustment to night work over consecutive shifts, as well as with shorter day sleeps and higher sleepiness. Based on early findings such as these, countermeasures were identified to help older shiftworkers cope with shift work (31). The recommendations included enhanced work time flexibility, increased recovery time, reduction of night work, rapidly rotating schedules and intensified medical surveillance.

The most widely discussed dimension of individual difference in circadian rhythm function is diurnal preference (also variously referred to as “chronotype” or “morningness versus eveningness”). An early field study of nurses, using physiological measures of circadian timing, found that morning types had more difficulties adjusting to nightwork (32). Subsequently, several studies have shown that among shiftworkers, morning types experience poorer sleep quality, particularly in relation to night shifts, and are at greater risk of developing shiftwork-related sleep disorders (33). Diurnal preference is commonly measured by self-report questionnaire. A scale developed by Torsvall and Åkerstedt (34) sought to address problems with earlier scales around psychometric quality and usability “in the field” (ie, length).

In recent years, evidence has begun to emerge of a potential genetic contribution to shiftwork tolerance and susceptibility to shiftwork related diseases (eg 35,).

## The development of epidemiological methods

The first epidemiological studies on shift work and long working hours often suffered from quality problems. The samples were insufficient for the detection of rare outcomes, and the study designs were cross-sectional, comparing shift workers to day workers who are often in higher socio-economic groups, and who experience different work characteristics and living habits. Exposure assessment was often crude, being sensitive to memory bias and giving imprecise information on the working hours patterns under study (5, 36, 37). The earlier epidemiological studies on working hours and health were also unable to take into account bias due to selection into shift work (the “healthy worker effect”), and suffered from insufficient control of confounders.

More recently, the use of registry-based working hour data based on daily pay-roll records has been a major step forward, as it provides precise information on exposure (17). It thereby reduces the risk of exposure misclassification and makes the results more relevant for formulating practical recommendations. Pay-roll records of working hours are not influenced by information bias, or attrition.

Other recent methodological developments in working hours and health research include experimental stepped-wedge pseudo-randomized trials and the use of the observational propensity score method, where data from large cohorts are clustered or matched in a way that mimics randomization (26, 38, 39). Some recent studies have employed case–crossover designs, which are essentially within-participant variations of case–control studies. These designs are most suited to situations where the effects on risks are immediate and transient, and the outcome is acute (27, 40). Longitudinal fixed-effects time-dependent modelling has also been used for the analysis of within-participant variation over time (41). The use of repeated exposure data also allows multiple baselines and adjustment for selection effects by excluding “diseased” shift workers who have switched to daywork (38).

## Shift work, cardiovascular diseases and cancer

The associations between shift work and cardiovascular diseases (CVD) have been extensively studied, with reviews of the topic being well cited (2, 42, 43). The associations between shift work and CVD are linked to the observed excess risks for some major risk factors of CVD: obesity (44), type-II diabetes (45), and hypertension (46). Following the classic study by Knutsson et al published in 1986 in the Lancet (47), which showed that the risk of CVD increased linearly with exposure to shift work of up to 20 years, scientists have subsequently sought to repeat the finding with different cardiovascular outcomes and determine exposure–response estimates. The use of registry-based exposure data has provided further evidence of the association between shift work, and incident coronary heart disease (48, 49) and stroke (50). However, the findings remain contradictory with respect to possible exposure–response relationship for night shifts (48) (49–51). This suggests that other working hour characteristics of shift work, in addition to night work, may contribute to the risk. The frequency of quick returns, which is linked to the direction and speed of shift rotation, is a strong candidate (49, 50). It should also be borne in mind that, since there are many pathways from shift work to CVD (43), simple unmoderated associations with, for example, the frequency of nights shifts, are improbable.

The International Agency for Research on Cancer (IARC) has twice concluded that night shift work is probably carcinogenic to humans (18). Their latest assessment in 2019 was based on strong mechanistic and sufficient animal evidence, while the epidemiological evidence in humans was judged to be limited. The epidemiological evidence was largely based on case–control studies (52) of high quality and with good exposure assessment, although evidence from cohort studies was less conclusive. The scientific community, as well as the IARC report, has repeatedly emphasized the need for better exposure assessment in all cancer studies (18, 36, 53). This requirement can be addressed relatively easily by case–control studies that assess exposure retrospectively. However, it is more problematic for prospective cohort studies, in which shift work exposure has been assessed many years previously through survey questions that do not adequately differentiate between different forms of work schedule. The newer pay-roll based cohorts have detailed shift work exposure metrics, but the short follow-up times that are currently available, and the lack of information on exposure prior to the baseline, limit their value in the investigation of the long-term health outcomes (54, 55).

## Countermeasures

Intervention studies examining countermeasures to tackle to the negative impact of shift work tend to fall into four categories: shift schedule design, controlled light exposure, behavioral and pharmacological approaches. Systematic reviews identified evidence favouring each of these, except for hypnotics in the pharmacological approaches. However, there was a lack of evidence of their effectiveness in countering the long-term health consequences of night work (56, 57).

In recent years, a key focus for research has been the importance of shift scheduling. For example, controlled intervention studies showed that fatigue is reduced in rapidly forward rotating shift schedules, as compared to slow, backward rotating schedules (58, 59). A literature review on shift scheduling, sleep and fatigue confirmed the findings related to speed and direction of rotation (12). Recently, these findings were confirmed in a large-scale longitudinal study, including more than 7700 participants (41). This study also observed increased fatigue and disturbed sleep in relation to quick returns.

The importance of designing night shift schedules according to scientifically based ergonomic principles was highlighted in a recent discussion paper (60). It was concluded that schedules that reduce circadian disruption may reduce cancer risk (particularly for female breast cancer) and that schedules that optimise sleep and reduce fatigue may reduce injury risk. Adherence to ergonomic principles of schedule design has also been shown to beneficially affect biomarkers of risk factors for ischemic heart disease (61).

Based on earlier research highlighting the key role of bright light as the main synchronizer of human circadian rhythms, many experimental studies have tested exposure to bright light to support the circadian adjustment to night work. In most cases these manipulations lead to improvements in, for example, subjective reports of sleep/wakefulness, wellbeing, and performance (36, 62, 63). However, it is notable that in one of the most well-controlled field studies (64), effects of light treatment were small and less than the effects of melatonin administration prior to bedtime. Unlike the field studies, which were largely unable to show effects on circadian timing, laboratory-based experiments simulating shift work under controlled conditions have managed to demonstrate enhanced adaption to night work (65).

Shift workers can be helped to mitigate the impact of their schedules through physical training (66) and other forms of health promotion. A field study demonstrated how a mobile app that provided tailored advice on managing sleep, fatigue and health behaviours improved fatigue and sleep outcomes among airline pilots experiencing circadian disruption as a result of working irregular flight schedules (67). A systematic review concluded that employer-led health promotion initiatives that are adapted to the needs of shift workers can be effective in supporting shift workers to lose weight and increase physical activity (68).

## Work time control

Work time control (WTC) (eg, self-rostering or participatory shift scheduling) is an important means of providing employees a better fit between their working hours and personal needs. WTC refers to the employees’ control over the duration and starting and finishing times of the working day / work shifts, the distribution of work days and days off, and when to take vacation (69). During the last decades, evidence has accumulated that WTC is beneficial for work–life balance, perceived health, promoting recovery and reducing the negative effects of work stress (70, 71). WTC may also moderate the effects of overtime work and reduce the adverse health consequences of long working weeks (72). In an overview of systematic reviews, the importance of interventions in both working time arrangements and WTC were emphasized (73).

There is a large variation in levels of WTC, depending on the type of working time arrangements that are in place, but shift workers usually report substantially lower control over their daily working hours than day workers (74). One of the earliest intervention studies in this area showed that self-rostering among nurses improved subjective health and recovery (75). However, recent quasi-experimental studies found that introducing participatory working time scheduling software in shift work had very few effects on perceived well-being and self-rated health, even though WTC, unit-level sickness absence, perceived work ability and sleep length all improved (39, 76, 77). A potential explanation of the lack of effect on well-being is that enhanced WTC may result in some employees choosing to work long shifts or other “unhealthy” working time arrangements that have been associated also with increased sickness absence (78). However, the same study also found that paying attention to good shift ergonomics when using participatory shift scheduling attenuated the negative effects on sickness absence.

## Long and short working hours

Our primary focus in this article up to now has been on shift and night work, reflecting the predominance of these topics within working hours research published in the Journal. However, research on the impact of long weekly working hours has also featured significantly in the journal’s history. Working long hours (eg, averaging ≥48 hours per week) prolongs exposure to physical and psychosocial stressors at work and may restrict opportunities for recovery.

There is a large body of evidence, as reflected in an early narrative review (3) and a subsequent systematic review (4), indicating that long working hours (defined in these reviews as >8 hours per day or >40 hours per week) are associated with a range of negative health outcomes that may reflect heightened stress exposure. These include depressive state, anxiety, truncated / disturbed sleep, and coronary heart disease. Another systematic review found that long working hours predicted weight gain and that they were a stronger predictor of weight-related outcomes than psychosocial factors (44). Most recently, evidence has emerged of an association between long working hours and all-cause mortality in a large, nationally representative cohort in China, with men and smokers being at the greatest risk (79).

Several studies have found that people working long hours tend to report more symptoms of psychological distress. However, they do not appear to be at greater risk of being clinically diagnosed with a common mental disorder (depression or anxiety-related disorders). A meta-analysis identified a small but significant association between long working hours (^3^55 hours per week) and depressive symptoms, although the effect was not significant when excluding cases of “psychological distress” (80). Two large population studies in Denmark found that longer weekly working hours (>40 hours and 48 hours per week) did not predict use of either psychotropic medication or psychiatric hospital treatment (81, 82), while similar conclusions were reached in an earlier study of senior hospital doctors (83). Though much less researched, there also seems to be no evidence to date to suggest that long working hours are associated with dementia (84).

While very long weekly working hours tend to be associated with increased accident risk, moderately long weekly hours do not. A systematic review identified a tendency for weekly working hours of > 55 hours to be associated with increased risk of safety incidents, while weekly hours of 41–54 hours were not (85). A population-based study based on register data found no association between working either >40 hours per week or > 48 hours per week, and accidental injury risk (24).

The health risks of long working hours can be reduced by working less. In recent years, there has also been interest in investigating the effects of shifting from 8- to 6-hour working days or to 4-day working weeks with 8-hour days. An early article (86) based on three controlled intervention studies, showed that a switch to a 6-hour day with retained salary decreased the incidence of musculoskeletal problems in the neck and shoulders. A later RCT study showed that shortening the working week reduced the occurrence of sleep problems, stress and fatigue (87). A systematic review identified evidence of improved wellbeing (88), at least in healthcare settings. However, there is lack of studies on clinically diagnosed health outcomes, sick leave and changes in productivity. It should also be pointed that part time work, which is reduced weekly working hours without retained salary, may not always be beneficial for health. A recent longitudinal study showed a higher risk of depression for workers with 15 hours of weekly working hours compared with full time workers, although the authors conceded that selection effects could not be completely ruled out (89).

## Working hours and health – what have we learned?

We have seen that the *Scandinavian Journal of Work, Environment & Health* has a rich history of publishing working hours and health research. Over the Journal’s 50 years, there has been a transition from mostly small-scale, cross-sectional field studies to large-scale epidemiological studies utilizing precise exposure assessment. This trend reflects the emergence of large register-based datasets and the development of analytic methods and innovative study designs, such as cluster-randomized controlled trials (90), data-mining research (91), within-participant variations of case–control studies, case-crossover designs and the use of observational propensity score methods to mimic randomization (26, 38, 39). The general quality of working hours research has greatly improved. The cumulative evidence from the original studies and reviews provides convincing evidence that shift work and long working hours, which are often associated with night work and insufficient recovery, increase the risk of poor sleep and fatigue, sickness absence and occupational injuries, and several chronic health conditions like CVD and cancer. The observed risks are strongly modified by individual and work-related factors, including the characteristics of the specific working hour patterns. Although the observed health risks of night shift work and long working hours are mostly low or moderate, the widespread prevalence of exposure and the hazardousness of the many associated potential outcomes makes such demanding working hours a major occupational health risk.

## Future research needs

Although we know much more about the health effects of shift work and demanding working hours today, the detailed exposure–response associations (where exposure is, for example, the number and combination of night shifts, long work shifts, quick returns, or other working time patterns) for acute, and especially chronic and rare health outcomes, are still mostly unknown. In the long run, cohort datasets that incorporate pay-roll data have the potential to clarify these exposure–response associations, especially if they are linked to adequate information on relevant confounders and mediators.

There is a need for further etiological research on potentially modifiable factors and to elucidate the underlying mechanisms linking demanding working hours with health disorders. Translation of the cumulative knowledge into effective countermeasures is also a necessity. To make this possible, we need large data sets (ie, larger sample sizes, multiple repeated measures, and longer exposure and follow-up periods) with good exposure assessment, along with strong study designs (92). In contrast to traditional intervention studies that are conducted at the group level, new epidemiological cohorts that include information on individual characteristics and changes in working hour patterns over time, offer the possibility to examine the impact of changes in working time arrangements at the individual level, and the development of personalized prevention strategies. In addition, the possibilities to link physiological, hormonal, genetic and immunological biomarkers to observational data may prove highly informative. The associations of demanding working hours with health often derive from a complex interaction of physiological, psychological and societal factors, highlighting the need for interdisciplinary research. Besides observational and experimental research, there is also a need for qualitative research and process evaluation of complex interventions based on the relevant guidance frameworks (93).

Lastly, we need to investigate the efficacy of implementations of health promotion practices and recommendations related to working time arrangements. Occupational health practitioners play a key role in many countries in tackling the health impact of demanding working hours. And yet there has been little systematic research to date on developing guidelines for medical surveillance (94), or for the implementation of the given recommendations (95).

We hope that these suggestions, as summarised in table 1, will guide and inspire future research in this important domain of occupational health research, and that the findings will continue to feature regularly in the pages of the Journal.

**Table 1:** Future research needs on working hours and health

	**Research priorities**	**Methodological needs**
**Cohort studies**	Chronic and rare health outcomes Potentially modifiable factors and pathways	Register-based exposure assessment Large sample sizes with repeated data and long exposure and follow-up periods Exposure–response associations of single and combined characteristics of working hour characteristics The use of biomarkers Data-mining research on previously non- defined working hour patterns
**Intervention ** **studies**	Acute and chronic health outcomes Changes in working hour characteristics, work-time control and health promotion The development of individualized working time patterns and combined prevention strategies Sleep and fatigue management Efficacy, effectiveness and implementation of countermeasures	Large samples, longer follow-ups Cluster-randomized controlled trials The use of observational propensity score methods to mimic randomization Analysis of subgroups like employees with health problems Interdisciplinary research Process evaluation
